# Keratocyte phenotype is enhanced in the absence of attachment to the substratum

**Published:** 2008-02-09

**Authors:** Martha L. Funderburgh, Mary M. Mann, James L. Funderburgh

**Affiliations:** UPMC Eye Center, Eye and Ear Institute, Ophthalmology and Visual Sciences Research Center, Department of Ophthalmology, University of Pittsburgh School of Medicine, Pittsburgh, PA

## Abstract

**Purpose:**

Keratocytes, mesenchymal cells populating the corneal stroma, secrete the unique transparent connective tissue of the cornea as well as opaque scar tissue after injury. Previous studies identified factors mediating keratocyte phenotype in vitro, particularly the expression of the keratan sulfate proteoglycans, which are essential for vision. Whereas earlier work emphasized effects of cytokines, the current study examines the effects of substratum attachment on keratocyte phenotype.

**Methods:**

Primary keratocytes from collagenase digestion of bovine corneas were cultured on tissue-culture plastic or on poly (2-hydroxyethylmethacrylate)(polyHEMA)-coated, non-adhesive surfaces. Secreted proteoglycans from culture media and cell-associated proteins were characterized using western blotting or isotopic labeling. Gene expression was characterized with quantitative reverse-transcriptase polymerase chain reaction (qRT–PCR). Secreted matrix was examined with immunostaining.

**Results:**

We observed that virtually all primary keratocytes participate in the formation of spheroidal aggregates, remaining viable for at least four weeks in vitro. Spheroid keratocytes secrete more keratan sulfate and keratocan than attached cells in the same culture medium. In spheroids, keratocytes accumulate substantial matrix in intercellular spaces, including keratan sulfate, lumican, keratocan, and collagens V and VI. The unattached cells undergo limited cell division and do not differentiate into myofibroblasts in response to transforming growth factor β (TGFβ), which is based on the expression of extra domain A (EDA) fibronectin and α-smooth muscle actin. Similarly, the platelet derived growth factor, a cytokine initiating the fibroblastic phenotype in attached keratocytes, had a limited effect on the spheroid-associated keratocytes. Ascorbate-2-phosphate was the only agent stimulating keratan sulfate secretion in the spheroid keratocytes.

**Conclusions:**

These results provide a new paradigm for understanding signals that regulate extracellular matrix secretion. For primary keratocytes, the alteration of the cellular environment in terms of cell-cell and cell-matrix interactions mediates and can override signals from soluble cytokines in influencing matrix expression and also in adopting other aspects of the fibroblastic and myofibroblastic phenotypes found in healing wounds.

## Introduction

Primary keratocytes of the corneal stroma synthesize the extracellular matrix crucial to the cornea’s functions of strength and transparency. When keratocytes in culture attach to a rigid substratum in a protein-free culture medium, the cells spread, develop extended dendritic processes, and maintain expression of keratan sulfate and keratocan, proteoglycan components uniquely expressed by differentiated keratocytes [1,2]. In the presence of serum or growth factors, attached keratocytes proliferate, adopt a fibroblastic morphology, and lose expression of keratocyte molecular markers [3,4]. We previously reported that a limited population of cells from bovine and human stroma can undergo extensive proliferation in vitro without losing the ability to adopt a keratocyte phenotype when cultured in low-mitogen conditions [5,6]. These cells expressed gene and protein profiles similar to embryonic corneal precursors and adult stem cells from other tissues. Unlike any other known cells, this population maintains an ability to adopt a keratocyte phenotype even after extensive population expansion in vitro.

In the presence of some growth factors, the stromal progenitor cells aggregate on the culture surface, forming spheroids, which subsequently detach from the substratum [5]. These spheroids maintained viability and were found to express high levels of keratocan mRNA and protein as well as keratan sulfate. Unlike monolayer cultures, the keratan sulfate accumulated in the intercellular spaces of the aggregated cells. Upon further study, we observed that some primary bovine keratocytes also exhibit a similar behavior in monolayer culture [5].

In the current study, we asked if the stromal cells participating in spheroid formation were limited to the progenitor population or if all keratocytes exhibited such behavior. We report that in the absence of substratum attachment, virtually all primary keratocytes remain viable and participate in spheroid formation. As unattached spheroids, keratocytes express higher levels of keratocan and keratan sulfate than cells maintained in the same culture medium in monolayer cultures. Several extracellular matrix components present in normal corneal stroma accumulate in the spheroids. In spheroids, the cells exhibit different responses to TGFβ and some other growth factors compared to substratum-attached cells.

**Figure 1:**
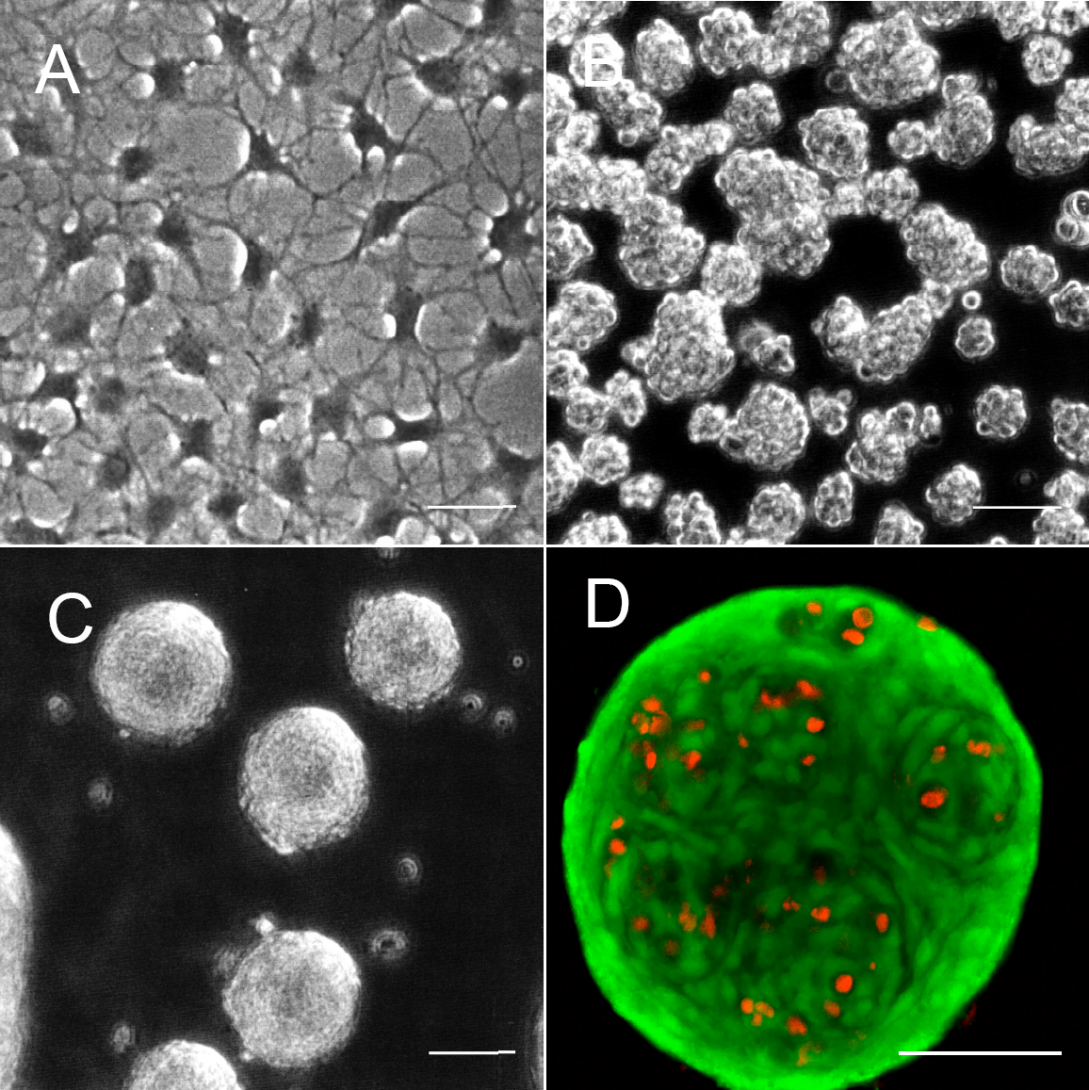
Spheroid formation by primary keratocytes. Freshly isolated primary bovine keratocytes were cultured in serum-free conditions on standard tissue culture plastic (**A**) or in vessels coated with polyHEMA, which prevented cell attachment (**B**,**C**,**D**). In two days (**B**), cells had formed aggregates, which condensed into smooth spheroids after two weeks of culture (**C**). **D**: In this panel, two-week spheroids were stained with vital dye Calcein AM to detect live cells (green) and propidium iodide (red) to detect dead cells. Scale bars show 50 µm.

## Methods

### Cell culture

Primary stromal cells were isolated from fresh bovine corneas by collagenase digestion, modified from a previously described procedure [2]. Fresh, whole bovine eyes (Pel-Freez, Rogers, AR) were rinsed with betadine then with CMF Saline-G (137 mM NaCl; 54 mM KCl; 1.0 mM Na_2_HPO_4_⋅7H_2_O; 1.1 mM KH_2_PO_4_; and 6 mM glucose; pH 7.2) containing antibiotics [2]. The corneas were excised, cutting about 2 mm inside the scleral rim. After two rinses in CMF-Saline G and one rinse in DF0 (DMEM/F12 with antibiotics [2]), the corneas were incubated in 1 mg/ml collagenase, type L (Sigma-Aldrich, St Louis, MO) in DF0 (2.5 ml/cornea) and shaken at 125 RPM for 1 h at 37 °C. The corneas were lightly scraped with a Cell Lifter (Fisher Scientific, Pittsburgh, PA) to remove remaining endothelium and epithelium then cut into 2 mm cubes. The minced corneas were digested as previously described for 1 h then rinsed five to six times in CMF-Saline G and once in DF0 followed by centrifugation at 50x g for 5 min and resuspension in the final digestion solution. After being placed overnight in ice, keratocytes were released by a final digestion in 10 mg/ml collagenase in DF0 with agitation for 2–4 h at 37 °C. Time and concentration of the final collagenase digestion was customized according to the manufacturer’s lot. The keratocyte suspension was filtered through a 70 µm cell strainer (Fisher Scientific, Pittsburgh, PA), and keratocytes were pelleted by centrifugation at 800x g for 10 min. The cell pellet was gently resuspended by tituration in DF0, and cells were washed twice more in DF0. The final yield was around 2 × 10^6^ cells per cornea.

Primary keratocytes were suspended at 2 × 10^5^ cells/ml in protein-free DF0 medium or in a serum-free medium which contains albumin, insulin, transferrin, and selenium (Advanced-DMEM [ADV]; Invitrogen, Carlsbad, CA) containing antibiotics and plated in tissue culture dishes precoated with FNC Coating Mix® (Athena Scientific, Nashua, NH) at 4 × 10^4^ cells/cm^2^ or were cultured under attachment-independent conditions (for spheroid formation) in tissue culture dishes coated with poly(2-hydroxyethyl methacrylate) (polyHEMA; Sigma-Aldrich) at 37 °C in a humidified atmosphere with 5% CO_2_. The polyHEMA-coated plates were prepared as described [7] with a concentration of 0.8 mg/cm^2^ in 95% ethanol before drying.

### Live-dead cell counting

Viable cells were labeled with the vital dye Calcein AM (Invitrogen) at 50 µg/ml for 15 min at 37° in culture medium. Propidium iodide (Sigma-Aldrich) at 5 µg/ml stained dead cells. Spheroids with their medium were transferred to a coverglass-bottomed dish (MatTek, Ashland, MA) and photographed immediately on a Bio-Rad Radiance Plus Laser Scanning Confocal microscope (Bio-Rad microscopy, Herfordshire, UK) using oil objectives at magnifications indicated in the figures. Quantification of live cells was performed by disaggregation of cell pellets in 0.5 ml TrypLE Express (Invitrogen), containing 50 µg/ml Calcein AM, for 20 min at 37 °C. In the final 5 min of the incubation, propidium iodide was added at 5 µg/ml. Cells were ticturated, centrifuged, resuspended in a small volume of medium, and counted manually for live (green) and dead (red) cells on a hemocytometer slide using a fluorescence microscope. Comparative cell growth was assessed using a fluorometric Alamar Blur assay (Accurate Chemical and Scientific, Westbury, NY) according to the manufacturer’s directions.

### Immunohistology

The cell spheroids are typically 80–100 µm in diameter, allowing them to be isolated by sedimentation. Fixing, staining, and washing steps are performed using the procedure previously described [5]. Primary antibodies were J19 (from Dr. Nirmala Sundarraj, University of Pittsburgh, Pittsburgh, PA) against keratan sulfate, Kera-C, affinity-purified goat antibody to the COOH-terminal peptide of keratocan (provided by Dr. Winston Kao, University of Cincinnati, Cincinnati, OH), monoclonal antibodies to lumican [8] (from Dr. Bruce Caterson, University of Cardiff, Cardiff, Wales), collagen V (Sigma-Aldrich), and collagen VI (Sigma- Aldrich). The spheres were fixed for 15 min in 3.2% paraformaldehyde in phosphate buffered saline (PBS), rinsed, then immunostained overnight at 4 °C. Secondary antibodies used for microscopy were goat anti-mouse IgG Alexa 545 and donkey anti-goat IgG Alexa 488 (Invitrogen). Staining of mitotic cells was performed after labeling the culture for 18 h with 10 μg/ml bromodeoxyuridine (BrdU) using immunological reagents provided by Roche Applied Science (Indianapolis, IN) according to the manufacturer’s directions.

**Figure 2:**
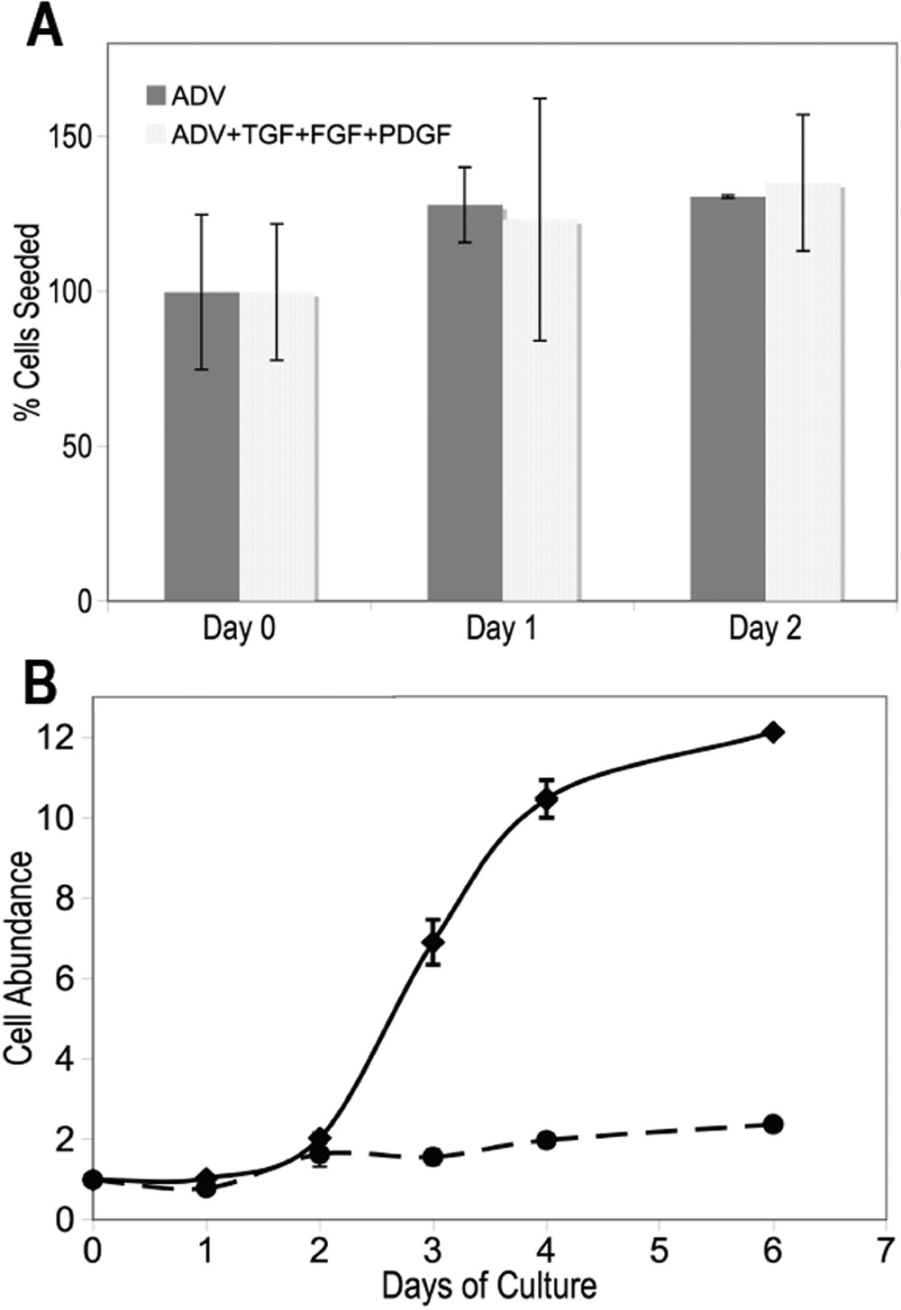
All keratocytes participate in sphere formation. **A**: Freshly isolated primary keratocytes were cultured in polyHEMA-coated dishes either in Advanced-DMEM (ADV, dark solid bars) or in Advanced-DMEM containing transforming growth factor beta-1 (TGFβ-1), fibroblast growth factor 2 (FGF2), and platelet derived growth factor BB (PDGF; light-stippled bars). Viable cells were labeled with Calcein AM, and the number labeled cells was determined by direct counting after trypsinization. Cell number is shown as a percentage of viable cells added to the original culture. **B**: Primary stromal cells were plated in Advanced-DMEM containing 10 ng/ml FGF2 under attachment conditions (solid lines) or as spheroids in polyHEMA-coated dishes (broken lines). Cells numbers were estimated by conversion of the dye Alamar Blue to a fluorescent form and normalized to the number of cells added to the culture. Error bars show the SD of triplicate analyses.

### Immunoblot analysis

Proteoglycans in a culture medium were purified by ion exchange chromatography, dialyzed against water, and lyophilized [3]. Samples normalized for cell number (by total cell DNA content) were separated on 4%–20% SDS–PAGE gels (Criterion, Bio-Rad Laboratories, Hercules, CA) followed by an electro-transfer to PVDF membranes. Keratan sulfate on the blots was detected by monoclonal antibody J19 [5], and keratocan was detected using goat polyclonal antibody, Kera-C, after the digestion of keratan sulfate with keratanase II and endo-β-galactosidase as previously described [4].

### Fluorophore-assisted carbohydrate electrophoresis (FACE)

Fluorophore-assisted carbohydrate electrophoresis (FACE) analysis of the components of keratan sulfate from cell culture media was performed as previously reported [4].

### Real-time quantitative polymerase chain reaction

Relative mRNA levels were analyzed by quantitative, real-time PCR (real time qPCR) as previously described [4]. Briefly, mRNA was isolated by RNeasy (Qiagen, Valencia, CA), treated with DNase-I (Ambion Inc., Austin TX), and precipitated with ethanol. cDNA was reversed-transcribed using SuperScript II (Invitrogen) with random primers. qPCR was performed in triplicate using an ABI7700 sequence detection system and analyzed with ABI software using a Δ-C_t_ method with rRNA as an internal standard for each cDNA sample. Primer sequences were reported previously [4].

**Figure 3:**
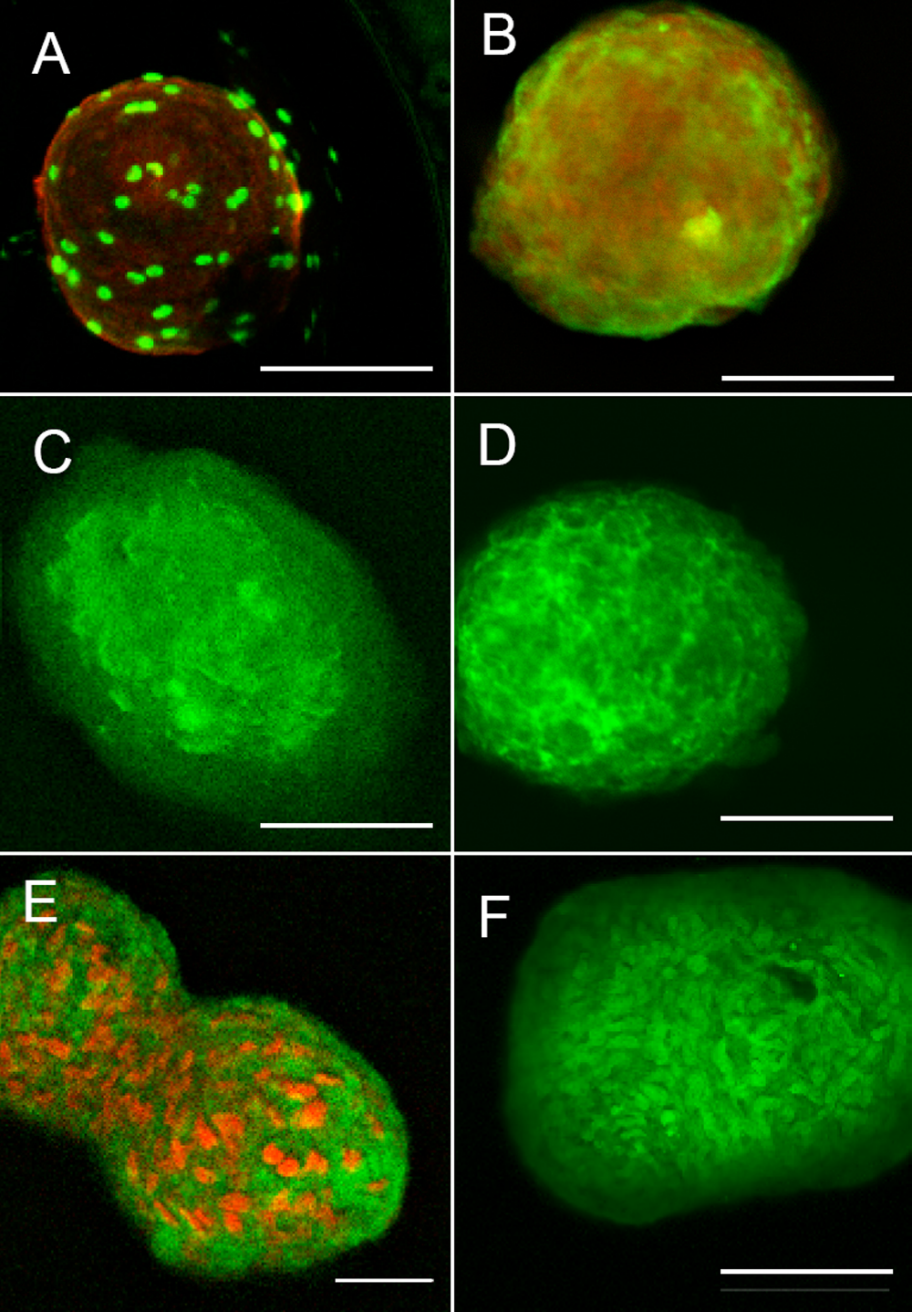
Proliferation and matrix synthesis by keratocytes in spheroids. **A**: Cell cycle status of keratocytes in spheroids was visualized by incorporation of BrdU (green) in spheroids after three days of culture in ADV medium containing FGF2 and PDGF. Cell nuclei were counterstained with propidium iodide (red). **B**: Keratan sulfate (green) and keratocan (red) were visualized after two weeks culture in ADV medium as described in Methods. **C**: Lumican was stained in spheroids after two weeks culture in ADV medium as described in Methods. **D**: Collagen I was stained in spheroids after two weeks culture in ADV medium as described in Methods. **E**: Collagen V (green) and nuclear stain (red) were stained in spheroids after two weeks culture in ADV medium as described in Methods. **F**: Collagen VI was stained in spheroids after two weeks culture in ADV medium as described in Methods. Bars show 50 μm.

## Results

We previously reported that progenitor cells from the corneal stroma cultured in a monolayer will form focal aggregates, which detach from the substratum and grow as spheroids [5]. The cells in the spheroids secreted stromal matrix proteins and expressed mRNA for keratocan, a unique product of differentiated keratocytes. Only a minority of the cells from any culture were involved. We initially sought to determine if all stromal cells participate in such attachment-independent growth or if spheroids can only be formed by progenitor cells, which make up less than 3% of the cells in bovine stroma. Aggregation of freshly isolated primary bovine keratocytes was examined in dishes coated with polyHEMA, a non-adhesive inert polymer [7]. As shown in Figure 1A, keratocytes in ADV, a low-mitogen serum-free medium, attach and adopt a dendritic morphology in tissue culture-treated plastic. In polyHEMA-coated dishes, the cells begin to aggregate after two days in culture (Figure 1B). Two weeks in non-attachment culture resulted in the keratocytes becoming highly compact spheroids 80–200 µm in diameter (Figure 1C). The staining of these spheroids with the vital dye, Calcein AM, (Figure 1D) demonstrated most of the cells to be viable. The outer cells of the spheroids appear to be flattened and parallel to the surface, and the inner cells are arranged in patterns of swirls, some suggesting the combining of multiple spheres. Dead cells, visualized by the incorporation of propidium iodide into the nucleus, were few and largely internal, consisting of less than 5% of the total.

**Figure 4:**
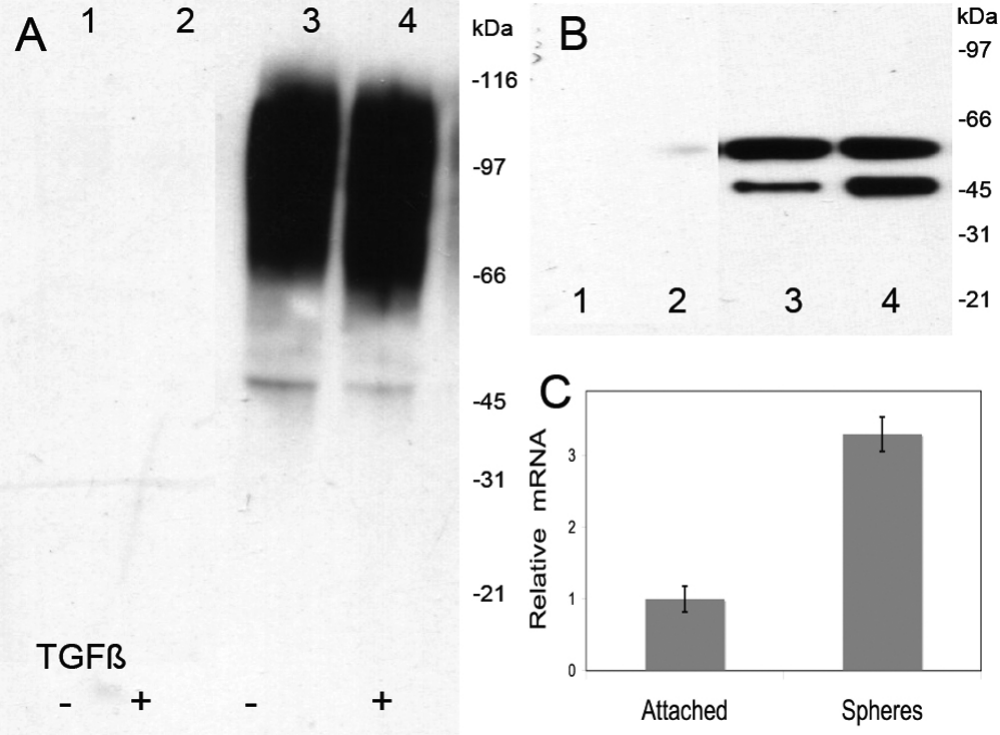
Production of keratan sulfate proteoglycans by spheroids. In **A** and **B**, primary keratocytes were cultured in Advanced-DMEM as attached cells (lanes 1 and 2) or as spheroids (lanes 3 and 4) in the presence (lanes 2 and 4) or absence (lanes 1 and 3) of TGFβ1 at 2 ng/ml for five days. Keratan sulfate proteoglycans from the culture medium were detected by immunoblotting using monoclonal antibody to keratan sulfate (**A**), and core protein keratocan was detected after digestion with endo-β-galactosidase and keratanase II as described under Methods (**B**). **C**: In this panel, the relative mRNA levels for keratocan in Advanced-DMEM for spheroids and attached cells were determined using qRT–PCR as described under Methods.

To determine what proportion of primary cells were incorporated into spheroids, a time course was performed documenting the total number of cells and their viability after freshly isolated primary keratocytes were cultured in substratum-independent conditions. An ADV serum-free medium (as in Figure 1) was compared to the medium supplemented with fibroblast growth factor 2 (FGF2), TGFβ1, and PDGF, factors that induce cell division in attached keratocytes. As shown in Figure 2A, the number of viable cells in the culture remained constant over a two-day period compared to the number of cells in the inoculum. The presence of cytokines had no effect on the cell number. This experiment suggests that all keratocytes participate in the formation of these substratum-independent spheroids and not just the cells with progenitor character.

**Figure 5:**
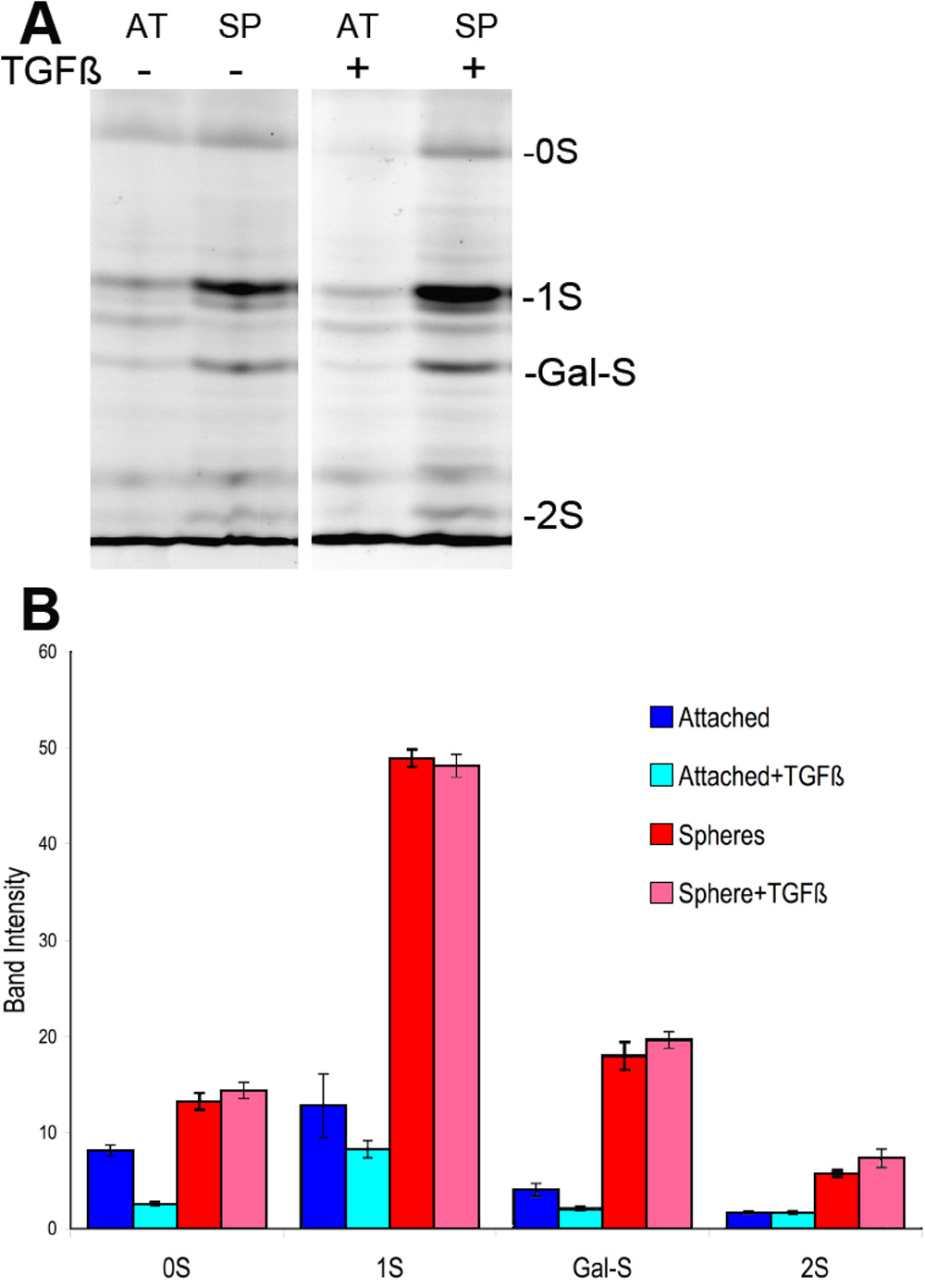
Fluorophore-assisted carbohydrate electrophoresis (FACE) analysis of keratan sulfate secreted by keratocytes. Keratan sulfate from culture medium was digested with keratanase II and endo-β-galactosidase, and the fragments were analyzed by fluorophore-assisted carbohydrate electrophoresis (FACE). **A**: Marked bands show products previously determined to be derived from bovine keratocyte keratan sulfate: 0S is the unsulfated disaccharide generated by endo-β-galactosidase; 1S is the monosulfated disaccharides generated by keratanase and endo-β-galactosidase; Gal-S is galactose sulfate, a product of keratanase digestion; and 2S is disulfated disaccharide, a product of keratanase digestion. Unmarked bands represent non-specific and/or unidentified products present in the dye or the digest. Keratan sulfate samples were isolated from the conditioned media of attached (AT) and spheroid (SP) cells cultured five days in Advanced-DMEM with (+) or without (-) TGFβ1 at 2 ng/ml. **B**: The chart shows the quantification of the keratan sulfate products in gels similar to that shown in **A**. Blue bars (left two of each group) are attached cells whereas red/pink (right two of each group) are from sphere cultures. The darker bars (Bars 1 and 3 of each group) are from cultures without TGFβ and the lighter bars (Bars 2 and 4 of each group) were treated with TGFβ. Error bars show standard deviation of triplicate analyses.

In a longer-term experiment shown in Figure 2B, the number of cells present in spheroids was compared to the number of cells cultured in an identical medium in monolayer culture. In the presence of FGF2, the number of attached cells increased by about 12 fold during a six-day incubation as determined by the fluorescent dye, Alamar Blue. However, under identical conditions, attachment-independent cells increased only slightly (about 40%). Adding PDGF to the medium with FGF2 did not stimulate increases in the number of cells in the spheroids (data not shown). The location of mitotic cells in spheroids was examined after three days by incorporating BrdU. As shown in Figure 3A, a small number of cells exclusively on the periphery of the spheres were seen to be dividing.

Keratocyte differentiation is characterized by the secretion of keratan sulfate proteoglycans, matrix components unique to the corneal stroma and essential for corneal transparency [9]. The expression of the keratan sulfate proteoglycan protein keratocan and its modification with the glycosaminoglycan keratan sulfate are volatile markers of keratocyte phenotype and are lost both in healing wounds and as the cells are cultured in fetal bovine serum [4,9,10]. When we compared keratan sulfate-containing proteoglycans in the conditioned media from attached and spheroid cultures, much higher levels of keratan sulfate were detected in the spheroid-conditioned medium (Figure 4A). With attached cells, we have previously found >90% of the keratan sulfate secreted into the culture medium. In the spheroids, significant amounts of keratan sulfate proteoglycan (KSPG) remained associated with the cells. As shown in Figure 3B, keratan sulfate (red) was abundant in the flattened layer of cells at the periphery and also detected in extracellular spaces between the internal cells. Keratocan (green) was present primarily in the intracellular spaces in the internal portion of the spheroids and also cell-associated. Lumican (Figure 3C), collagen I (Figure 3D), collagen V (Figure 3E), and collagen VI (Figure 3F), extracellular matrix components of normal corneal stroma, were also detected in the spheroids.

The addition of TGFβ to the spheroid cultures did not (as previously documented for attached cultures) reduce the abundance of keratan sulfate (Figure 4A, lane 4). Keratocan was also increased in the spheroid-conditioned medium compared to the medium from attached cells (Figure 4B). After five days of culture, mRNA levels for keratocan were increased three to five fold in spheroids compared to mRNA from attached keratocytes (Figure 4D).

Fragments of keratan sulfate chains after digestion with specific glycosidases were analyzed using FACE gels; these results are shown in Figure 5. This technique allows the detection of three disaccharides as well as galactose–sulfate [4]. Each of these components showed stronger bands in keratan sulfate produced by spheroids compared to that secreted by a similar number of attached cells. Quantitative analysis of the bands (Figure 5B) showed that the sulfated components (1S, GalS, 2S) increased more than the unsulfated disaccharide (0S). Treatment of attached keratocytes with TGFβ is known to reduce the keratan sulfate they secrete [4]. The analysis in Figure 5 confirmed these findings. Keratan sulfate from spheroids, however, was not decreased by TGFβ treatment, and in fact, the disulfated disaccharide (2S) was increased in response to TGFβ. The amount of changes in keratan sulfate fragments in response to TGFβ treatment is summarized in Table 1. In attached cells, total keratan sulfate was decreased by about half with the greatest change observed in unsulfated (0S) disaccharides. In keratan sulfate secreted by spheres, no change in overall keratan sulfate was observed, but a 28% increase in the disulfated disaccharide (2S) was found.

**Table 1:** Keratan sulfate composition changes in response to TGFβ.

	**Attached**	**Sphere**
0-S	32**	109
1-S	65**	98
Gal-S	50**	109
2-S	98	128*
Total	54**	104

Keratan sulfate fragments (named as in Figure 5) from attached and sphere cultures were compared with and without treatment with TGFβ (as in Figure 5). Relative abundance of fragments in treated cultures is given as a percentage of the same fragment in control (untreated) cultures. The asterisks indicate a significant reduction in keratan sulfate fragments in response to TGFβ treatment in triplicate samples using Student's t-test. The single asterisk indicates a p<0.05 and the double asterisks indicate a p< 0.01.

**Figure 6:**
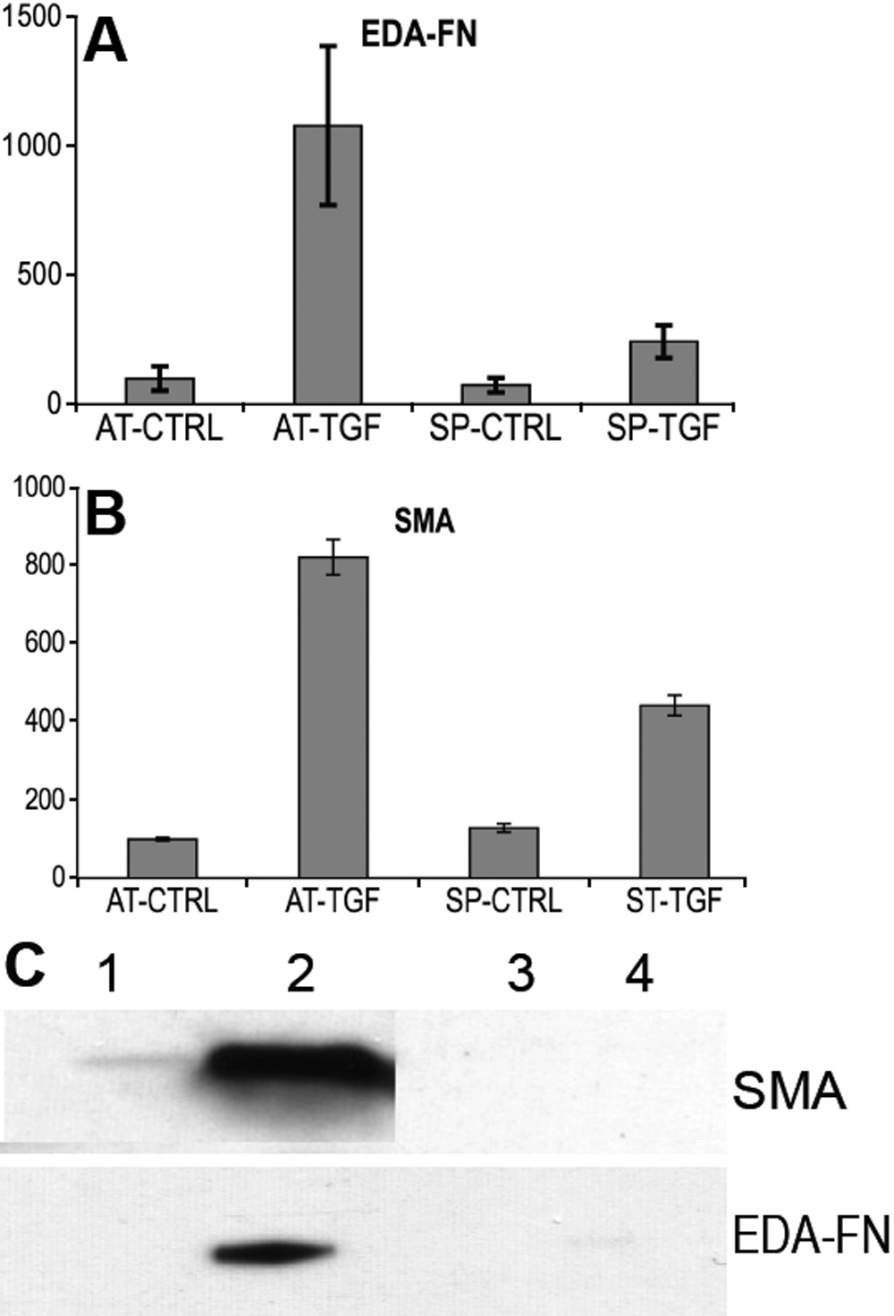
Expression of fibrotic markers in spheroids in response to TGFβ. Cells in Advanced-DMEM were cultured as attached cells (AT) or spheroids (SP) with (TGF) or without (CTRL) TGFβ (2 ng/ml for five days). The relative abundance of mRNA was determined for the EDA form of fibronectin (EDA-FN; **A**) and for alpha-smooth muscle actin (SMA; **B**). **C**: Immunoblotting determined protein abundance in lysed samples of the spheroids for smooth muscle actin and EDA-fibronectin. Lane 1, attached control; Lane 2, attached TGFβ-treated; Lane 3, spheroid control; Lane 4, spheroid, TGFβ-treated.

An altered response of keratocytes to TGFβ in spheroids was also detected in the characteristic expression of genes involved in the fibrotic-myofibroblastic transition of the keratocyte. As shown in Figure 6, alpha smooth muscle actin mRNA, measured by qRT–PCR, was found to be upregulated 8–10 fold in attached keratocytes in response to TGFβ. In the spheroids, alpha-smooth muscle actin mRNA increased but only by four to fivefold. Similarly, the EDA splice-form of cellular fibronectin is upregulated more than 10 fold in attached cells by TGFβ, but in spheroids, the mRNA increased by less than threefold. Immunoblot detection of these two proteins showed even more marked differences in their expression levels. Smooth muscle actin and EDA-fibronectin were abundant in cells layers of attached cells after TGFβ treatment but could not be detected in spheroids with or without TGFβ treatment.

**Figure 7:**
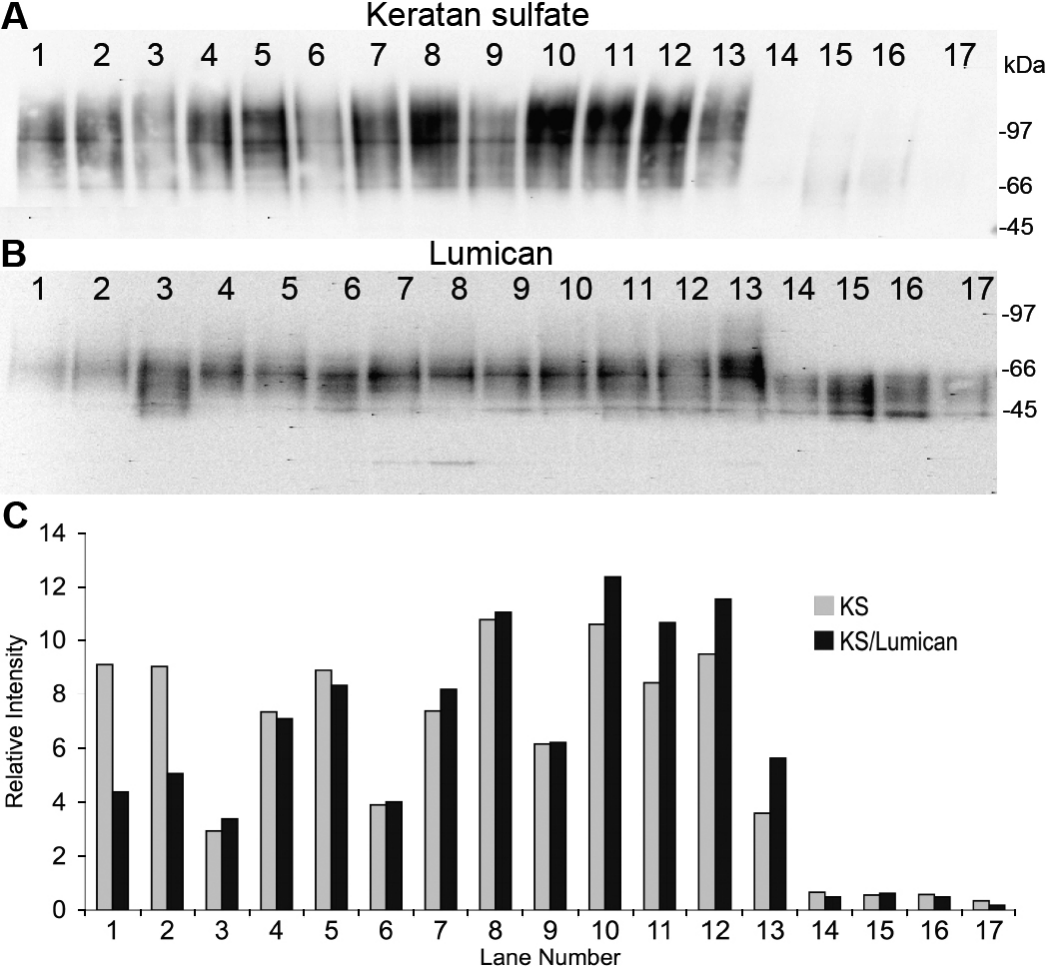
Keratan sulfate secretion in spheroids as a function of culture conditions. Keratan sulfate was detected by immunoblotting directly from conditioned media after five days of culture of primary spheroids in protein-free media. The first 13 samples were cultured in protein-free DME/F12. Additions were: 1. None; 2. Insulin, transferrin, selenium (ITS); 3. FGF2, 10 ng/ml; 4. TGFβ1, 2 ng/ml; 5. PDGF BB, 10 ng/ml; 6. TGFβ1 and FG2; 7. ITS and FGF2; 8. ITS and TGFβ1; 9. ITS, TGFβ1, and FGF2; 10, ascorbate 2 phosphate 100 μM (A2P); 11. A2P and ITS; 12. A2P and TGFβ;13. A2P and FGF2; 14. keratinocyte serum free medium (KSFM); 15. KSFM with A2P; 16. KSFM with ITS; and 17. KSFM and TGFβ1. In **B,** the immunoblot shown in **A** was stripped and reprobed for lumican. Because of its modification with keratan sulfate, lumican appears as a heterogeneous smear and not as a sharp band. **C**: This panel shows quantitation of the keratan sulfate from **A** (gray bars) and the ratio between keratan sulfate and lumican from **A** and **C** (black bars).

Several previous studies as well as the results in Figure 5 indicate that keratan sulfate secretion by primary keratocytes in attachment cultures is highly responsive to growth factors and serum. Figure 7 presents results of a survey of growth factors by immunoblotting keratan sulfate directly from a conditioned medium. In Figure 7B, the blot was stripped and reprobed with an antibody to lumican, a KSPG core-protein less responsive to growth factors than keratan sulfate or keratocan. The ratio of keratan sulfate to lumican (Figure 7C; black bars) showed similar variation to that of the keratan sulfate alone (gray bars). This illustrates that keratan sulfate is specifically regulated in these cultures. The ascorbate analog, ascorbate-2 phosphate, generated the highest levels of keratan sulfate (lanes 10,11,12). FGF2, a growth factor previously reported to stimulate keratocan and keratan sulfate in attached cells [2], did not stimulate keratan sulfate in spheroids (Lane 3 and 13). Insulin, another cytokine reported to support keratocyte phenotype, had a marginal effect on keratan sulfate secretion (lanes 2 and 11). PDGF and TGFβ, growth factors that convert attached keratocytes to fibroblasts thereby reducing keratan sulfate synthesis, similarly had little effect on keratan sulfate synthesis by attachment-independent keratocytes (lanes, 5,6,8,9,12). Keratocytes cultured in a low calcium medium (lanes 14–17), which was recently reported to maintain a differentiated keratocyte phenotype in vitro [11], remained viable (not shown) but secreted little keratan sulfate.

## Discussion

We originally reported attachment-independent spheroids generated from stromal progenitor cells and primary keratocytes when they were cultured as monolayers in the presence of FGF2 without serum [5]. Under those conditions, only a small fraction of the cells detached from the substratum. Spheroids are also formed by neural stem cells in the presence of FGF2 [12]. Such spheroids appear to be generated by clonal expansion of single stem cells [13]. Spheres, similar to those of bovine progenitor cells, have also been reported to arise in suspension cultures of mouse corneal stromal cells [14]. However, the spheroids formed by keratocytes and stromal progenitor cells appear to differ fundamentally from neural stem aggregates (known as neurospheres). Neurospheres express gene characteristics of neural progenitor cells [15], whereas spheres formed from bovine progenitor cells [5], suspended mouse stromal cells [14], and differentiated keratocytes (this study) all express mRNA and proteins typical of differentiated keratocytes. As shown in Figure 3 and Figure 4, aggregates contain high levels of mRNA and proteins that are abundant in adult stroma. We have also observed that genes expressed by progenitor cells, such as *PAX6,* *SIX2,* and *SIX3*, are not expressed in the aggregates (data not shown). Thus, unlike neurospheres, spheroids from stromal cells appear to induce or maintain cells in a differentiated phenotype.

An important observation of the current study is that essentially all of the viable cells from the stroma participate in forming these bodies (Figure 2). Thus, the formation of attachment independent spheroids appears to be a property of keratocytes and not of progenitor cells. In fact, the expression of differentiated markers in progenitor spheroids suggests that attachment-independent growth induces differentiation to keratocytes. Such a conclusion is strongly supported by our recent finding that the culture of human stromal stem cells as an attachment-dependent pellet induced expression of an array of keratocyte-specific genes and downregulated the expression of several stem cell marker genes [16].

The observation that keratocyte-based spheroids differ from the neurosphere type of bodies formed by stem cells does not mean that this behavior is unique. Several cultured cells form aggregates when deprived of substratum attachment. In some cases, like keratocytes, aggregation into spheroids results in stable viable populations of cells expressing a high level of differentiated function. Substratum-free cultures of hepatocytes [17], chondrocytes [18], cardiomyocytes [19], and pancreatic acinar cells [20] have all been described. We recently showed that human corneal stromal stem cells, when cultured as substratum-free pellets, adopted gene expression patterns characteristic of differentiated keratocytes and secreted an abundant extracellular matrix [16].

The characteristics of the substratum-independent cultures provide new insights into the interplay between the cellular environment and differentiated phenotype. Ever since Conrad [21,22] showed in 1974 that cells from the corneal stroma lose keratan sulfate synthesis in vitro, studies have attempted to define culture conditions under which keratocytes can maintain a phenotype similar to that in vivo. Funderburgh et al. [23] showed that in serum-containing media, keratocytes secreted keratan sulfate proteoglycan core proteins, but these proteins were modified with short undersulfated keratan sulfate chains. In 1999, Beales et al. [1] found that primary cultures of keratocytes in protein-free or low-mitogen media produced more keratan sulfate and keratan sulfate proteoglycans with higher molecular weight than those in serum-containing media. In subsequent studies, growth factors PDGF and TGFβ, were observed to down-regulate the size and amount of keratan sulfate in cultures of primary keratocytes, and FGF2 was found to upregulate keratan sulfate, even in dividing cells [2]. More recently, Musselman et al. [24] reported insulin to be supportive of keratocyte differentiated characteristics in vitro. Thus, the expression of keratocyte phenotypic characteristics has largely been characterized in terms of responses to soluble cytokines and growth factors.

The current study adds a new dimension to our understanding of the maintenance of keratocyte phenotype. In the absence of attachment to a rigid substratum, keratocytes produce more keratan sulfate and keratocan than attached cells in the same media (Figure 4). This was seen both in protein-free media (DF0) and in a medium with albumin and insulin (ADV) (Figure 5A). Growth factors (PDGF and TGFβ) that were well established to abrogate the expression of keratocyte phenotype in attached cultures [3,4,25,26] had little effect on keratocan and keratan sulfate synthesis in attachment-independent cells. Alternately, two growth factors, FGF2 and insulin, that were reported to maintain aspects of the phenotype [2,24] also showed little effect (Figure 7). In addition to the reduced response of keratan sulfate and keratocan to growth factors, keratocytes showed a marked reduction in cell division when in substratum-free culture (Figure 2) as well as a reduction in the expression of genes and proteins associated with the myofibroblast phenotype, EDA-fibronectin, and alpha-smooth muscle actin (Figure 7). Conversely, a culture in reduced calcium, a condition well known to disrupt cell-cell junctions, abrogated keratan sulfate synthesis even in the presence of growth factors (Figure 7). The implication of these results is that cell-cell interactions are important perhaps essential for keratocyte differentiation, whereas cell-matrix interactions may be necessary for response to mitogens.

The abundant extracellular matrix in the keratocyte spheres (Figure 3) demonstrates that there is no lack of substratum for cell-matrix interactions. The major differences between the cellular environment for cells in spheroids and attached cultures appear primarily to be the lack of a rigid substratum and the greatly increased cell density in the sphere cultures. The presence of a rigid substratum has been shown to increase response to TGFβ in the expression of smooth muscle actin [27] and may be the reason that spheroid keratocytes produce little smooth muscle actin compared to attached cells. It is not immediately obvious, however, that flexibility of substratum would account for the other responses of the keratocytes in spheroids or that the spheroids model the corneal stroma in this regard.

A more likely reason for the phenotypic response of keratocytes in spheroids is the extremely high density of cells in these bodies. Increased density allows greater communication among the cells either via secreted factors or by direct cell-cell interactions. Stromal cells in culture have cell-cell junctions using both connexin and cadherin proteins [28,29]. Gap junctions are known to support the differentiated phenotype of several cell types and are present in stromal cells in vivo [30-34]. Thus, we hypothesize that an increase in cell-cell junctions may provide signals mediating phenotypic enhancement of keratocytes in substratum-free conditions. This hypothesis is supported by the recent demonstration in our laboratory that free-floating pellet cultures of corneal stromal stem cells differentiate into keratocyte-like cells and exhibit an increase in the number of cadherin-11 and connexin-containing cell–cell junctions [16]. It is clear that further analysis will be required to confirm this speculation. However, the current study provides a new experimental system for defining the role of the extracellular environment on keratocyte phenotype and of the control of biosynthesis of an important class of proteoglycans.
